# Akinetopsia as epileptic seizure^☆^

**DOI:** 10.1016/j.ebcr.2013.04.002

**Published:** 2013-05-20

**Authors:** Kotaro Sakurai, Tsugiko Kurita, Youji Takeda, Hideaki Shiraishi, Ichiro Kusumi

**Affiliations:** a Department of Psychiatry and Neurology, Hokkaido University School of Medicine, Sapporo, Japan; b Department of Pediatrics, Hokkaido University School of Medicine, Sapporo, Japan

**Keywords:** Akinetopsia, Epilepsy, Epileptic seizures

## Abstract

Akinetopsia is a rare syndrome in which a patient specifically loses the ability to perceive visual motion following bilateral cortical lesions outside the striate cortex. We describe a patient who showed akinetopsia recurrently as epileptic seizures.

The patient was a 61-year-old man. At age 46, a cerebral arteriovenous malformation in the right parietal lobe was discovered. At age 58, he began to have a recurrent visual symptom by which smooth movements of objects suddenly appeared, resembling freeze frames in a motion picture. This symptom was paroxysmal and recurrent. Both EEG and magnetoencephalography showed repetitive right temporal spikes. We diagnosed his visual symptom as akinetopsia, which was aroused by hyperexcitability of the right temporal and parietal cortices, including area MT/V5. We administered carbamazepine 200 mg/day, which suppressed his akinetopsic symptom completely.

## 1 Introduction

Akinetopsia is a rare syndrome in which a patient specifically loses the ability to perceive visual motion following bilateral cortical lesions outside the striate cortex [1].

Patients with akinetopsia say that smooth movements of objects appear as a discontinuous freeze frame image [2]. Therefore, patients have difficulty, for example, in pouring tea into a cup because the fluid appears to be frozen, like a glacier [3]. The symptom is believed to result from damage to the visual motion pathway, especially area MT/V5 [1]. Transcranial Magnetic Stimulation study revealed that akinetopsia can be induced selectively and temporarily by magnetic stimulation of area MT/V5 in healthy subjects [4]. Akinetopsia was also reported in patients with traumatic brain injury, Alzheimer's disease, and stroke [2], [5], [6]. This report describes a patient who showed akinetopsia recurrently as epileptic seizures.

## 2 Case report

**Fig. 1:**
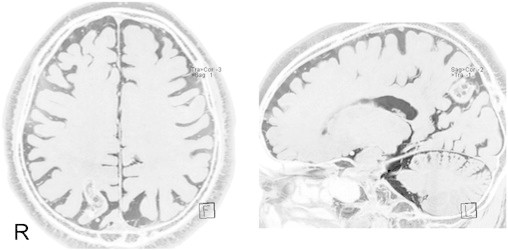
Magnetic resonance imaging (CISS). The lesion is visible in the right parietal lobe.

**Fig. 2:**
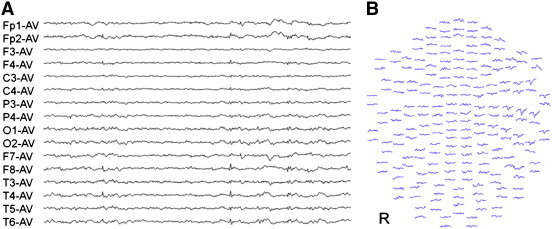
(A) Interictal. electroencephalograhy (EEG) EEG spikes are visible in the right temporal area. (B) Waveforms of MEG spikes recorded in all 204 channels. The MEG spikes appear in the right temporal area.

**Fig. 3:**
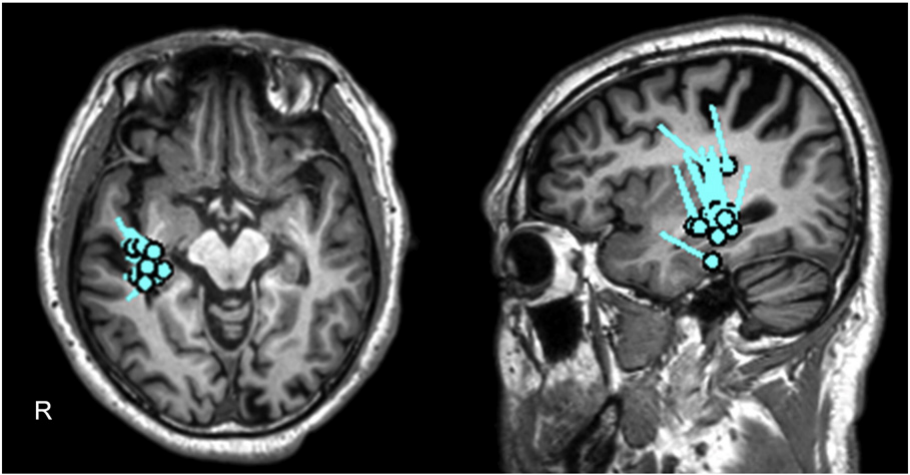
Equivalent current dipoles calculated from the MEG spikes. Equivalent current dipoles are clustered in the right mesial temporal lobe.

The patient was a 61-year-old right-handed Japanese man. His psychomotor development was normal. At age 46, a cerebral arteriovenous malformation (AVM) in the right parietal lobe was discovered. Although radiotherapy was performed to the AVM at our hospital, it did not result in the complete obstruction of the nidus. He had occasionally experienced visual hallucinations of flashes in the left visual field from the age of 47 years. Although this disappeared several years later, at age 58, he began to have another visual symptom by which smooth movements of objects suddenly appeared, resembling freeze frames in a motion picture. If the symptom occurred during a conversation, words were heard at normal speed, but the figure of the person halted, as in a freeze frame of a movie. Shape, size, color vision, and visual fields were normal. The symptom appeared in the entire visual field. This symptom appeared subsequent to being aware of a bad feeling and lasted for several seconds. When no object moved in the view during the symptom, it did not appear to freeze. However, the feeling that the whole view was shaking appeared. This symptom occurred several times per month. Although the patient underwent reexamination for AVM at our hospital, the nidus was obstructed completely. Because the symptom came to occur several times each day at age 59, he was referred to the Department of Neurosurgery at our hospital. The visual symptom was diagnosed as an epileptic seizure, and treatment with antiepileptic drugs was started. However, valproate and levetiracetam failed to control his seizures. At age 61, he was referred to our department. He said about his seizures, “Suddenly, I was feeling sick; then, I would go into the world like freeze frames several times a day.” His neurological examination was normal. Brain MRI revealed a high-intensity area suggestive of AVM in the right parietal lobe (Fig. 1). Scalp interictal EEG and magnetoencephalography (MEG) showed repetitive right temporal spikes (Fig. 2). Equivalent current dipoles (ECDs) calculated from these MEG spikes were clustered in the right mesial temporal lobe (Fig. 3). We diagnosed his visual symptom as akinetopsia, which was aroused by the hyperexcitability of the right temporal and parietal cortices, including area MT/V5. We administered carbamazepine 200 mg/day, which suppressed his akinetopsic symptom completely. The right temporal spikes on EEG had disappeared. He has continued CBZ and has been living since with no symptoms.

## 3 Discussion

We describe a patient who showed akinetopsia as epileptic seizures. The akinetopsic symptom was paroxysmal and recurrent. Both EEG and MEG showed definite abnormalities. Furthermore, the symptom disappeared with adequate antiepileptic drug therapy.

In our case, the cerebral lesion was detected in the right parietal lobe and spared area MT/V5. However, interictal EEG and MEG showed hyperexcitability in the right temporal area. Equivalent current dipoles calculated from MEG spikes were clustered in the right mesial temporal lobe, and the autonomic symptom at the time of clinical seizure onset also suggests the involvement of mesial temporal structures [7]. Because of the lack of ictal EEG record, we were unable to determine the ictal onset zone of the seizures. The epileptic discharges presumably spread to the right mesial temporal and temporo-parietal area containing MT/V5 judging by the clinical symptoms and interictal EEG and MEG. Therefore, akinetopsia in our case might be explained by the transient dysfunction of area MT/V5 by epileptic discharges in addition to the right parietal lesion.

Reportedly, bilateral cortical injury might be necessary for symptomatic akinetopsia [1], [5], [6]. However, Cooper et al. reported that unilateral lesions can induce global akinetopsia as well [2]. Similarly, both a lesion and the electroencephalogram abnormality were detected only in the right hemisphere in our case. Therefore, we inferred that even a unilateral pathological change might cause akinetopsia. However, because ictal EEG was not recorded, the possibility that the epileptic discharge had spread to both hemispheres during the seizures cannot be denied.

In conclusion, akinetopsia can present as an epileptic seizure. Hyperexcitability of the right temporo-parietal cortex might cause akinetopsia.
